# Bis[1,3-bis­(diphenyl­phosphan­yl)propane]­copper(I) tetra­chlorido­gallate(III)

**DOI:** 10.1107/S1600536812024269

**Published:** 2012-06-02

**Authors:** Nian-Nian Wang, Feng Hu, Tai-Ke Duan, Qun Chen, Qian-Feng Zhang

**Affiliations:** a Institute of Molecular Engineering and Applied Chemistry, Anhui University of Technology, Ma’anshan, Anhui 243002, People’s Republic of China; b Department of Applied Chemistry, School of Petrochemical Engineering, Changzhou University, Jiangsu 213164, People’s Republic of China

## Abstract

In the title compound, [Cu(C_27_H_26_P_2_)_2_][GaCl_4_], the Cu^I^ atom in the complex cation is *P*,*P*′-chelated by two 1,3-bis­(diphenyl­phosphan­yl)propane ligands in a distorted tetra­hedral geometry, while the Ga^III^ cation is coordinated by four chloride anions in a distorted tetra­hedral geometry. In the crystal, weak C—H⋯π inter­actions occur between adjacent complex cations.

## Related literature

For background to copper(I) phosphane compounds, see: Bownaker *et al.* (1995 ▶); Nicola *et al.* (2005 ▶); Lobana *et al.* (2009 ▶). For related structures, see: Xie *et al.* (1997 ▶); Comba *et al.* (1999 ▶); Rudawska & Ptasiewicz-Bak (2003 ▶).
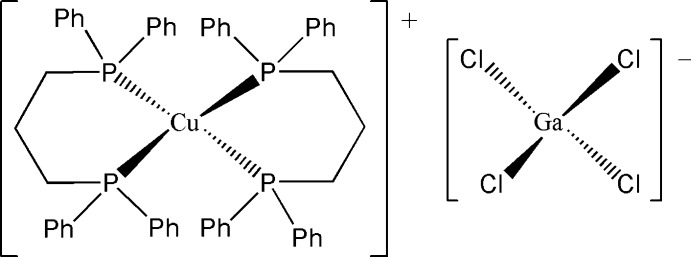


## Experimental

### 

#### Crystal data

[Cu(C_27_H_26_P_2_)_2_][GaCl_4_]
*M* *_r_* = 1099.90
Monoclinic, 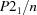

*a* = 21.077 (4) Å
*b* = 11.200 (2) Å
*c* = 22.605 (5) Å
β = 99.424 (3)°
*V* = 5264.3 (18) Å^3^
*Z* = 4
Mo *K*α radiation
μ = 1.28 mm^−1^
*T* = 296 K
0.40 × 0.25 × 0.09 mm

#### Data collection

Bruker SMART APEXII CCD area-detector diffractometer
Absorption correction: multi-scan (*SADABS*; Bruker, 2001 ▶) *T*_min_ = 0.629, *T*_max_ = 0.894
32381 measured reflections
12058 independent reflections
6644 reflections with *I* > 2σ(*I*)
*R*_int_ = 0.052

#### Refinement

*R*[*F* ^2^ > 2σ(*F* ^2^)] = 0.055
*wR*(*F* ^2^) = 0.159
*S* = 1.04
12058 reflections
577 parameters
H-atom parameters constrained
Δρ_max_ = 0.85 e Å^−3^
Δρ_min_ = −0.76 e Å^−3^

### 

Data collection: *APEX2* (Bruker, 2007 ▶); cell refinement: *SAINT* (Bruker, 2007 ▶); data reduction: *SAINT*; program(s) used to solve structure: *SHELXS97* (Sheldrick, 2008 ▶); program(s) used to refine structure: *SHELXL97* (Sheldrick, 2008 ▶); molecular graphics: *SHELXTL* (Sheldrick, 2008 ▶); software used to prepare material for publication: *SHELXTL*.

## Supplementary Material

Crystal structure: contains datablock(s) I, global. DOI: 10.1107/S1600536812024269/xu5551sup1.cif

Structure factors: contains datablock(s) I. DOI: 10.1107/S1600536812024269/xu5551Isup2.hkl

Additional supplementary materials:  crystallographic information; 3D view; checkCIF report

## Figures and Tables

**Table 1:** Hydrogen-bond geometry (Å, °) *Cg*1 and *Cg*2 are the centroids of the C21–C26 and C81–C86 benzene rings, respectively.

*D*—H⋯*A*	*D*—H	H⋯*A*	*D*⋯*A*	*D*—H⋯*A*
C14—H14⋯*Cg*1^i^	0.93	2.77	3.702 (8)	175
C55—H55⋯*Cg*2^ii^	0.93	2.66	3.526 (5)	155

Symmetry codes: (i) 
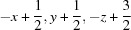
; (ii) 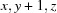.

**Fig. 1.**
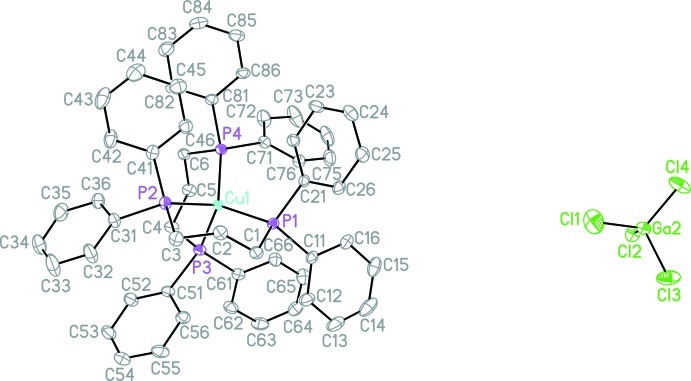
Perspective view of the title compound with displacement ellipsoids at the 50% probability level.

## supplementary crystallographic information

### Comment

There are a number of published studies of solution equilibria and structures that involve copper(I) compounds with phosphane ligands with copper(I)-to-ligand ratios (Bownaker *et al.*, 1995). Mononuclear phosphane-copper(I) complexes with chelating and bridging phosphine ligands in various coordination modes have been well isolated and structurally characterized (Lobana *et al.*, 2009). For examples, copper(I) nitrate and halide complexes of stoichiometry Cu(dppm)X (dppm = bis(diphenylphosphanyl)methane), Cu_2_(dppe)_3_X_2_ (dppe = bis(diphenylphosphanyl)- ethane), Cu(dppe)_2_X, and Cu(dppp)X (dppp = bis(diphenylphosphanyl)propane) (X = NO_3_, Cl, Br, and I) have been prepared and structurally characterized (Nicola *et al.*, 2005; Comba *et al.*, 1999; Xie *et al.*, 1997). It appears that the copper(I) complexes could be stabilized by organic phosphane ligands. Herein, we reported that an anionic complex, [Cu(dppp)_2_][GaCl_4_], with tetrahedral copper(I) in the [Cu(dppp)_2_]^+^ cation and tetrahedral gallium(III) in the [GaCl_4_]^-^ anion.

The title compound crystallizes in the monoclinic space group *P*2_1_/*c*. The molecular structure consists of the cationic [Cu(dppp)_2_]^+^ unit and the anionic [GaCl_4_]^-^ unit (Fig.1). The central copper(I) atom is coordinated by four phosphorus atoms from two dppp ligands. The strain of six-membered chelating ring is observed from the two low P—Cu—P bond angles of P1—Cu1—P2 = 99.18 (4)° and P3—Cu1—P4 = 98.34 (4)°, compared to the normal bond angle of 109°. The CuP_2_C_3_ skeleton is not planar because of the distorted tetrahedrally coordinated copper atom with the average Cu—P bond length of 2.3168 (11) Å, which is similar to that found in [Cu(dppp)_2_][ClO_4_] (Xie *et al.*, 1997) and [Cu(dppp)_2_][BF_4_] (Comba *et al.*, 1999). In the tetrahedral [GaCl_4_]^-^ anion, the average Ga—Cl bond length is 2.152 (2) Å and the average Cl—Ga—Cl bond angles is 109.45 (10)°, which are compared with those in the orthorhombic [Bu_4_N][GaCl_4_] salt (av. Ga—Cl = 2.169 (2) Å and av. Cl—Ga—Cl = 109.9 (1)°) (Rudawska & Ptasiewicz-Bak, 2003).

### Experimental

To a suspension of CuCl (75 mg, 0.75 mmol) in CH_3_CN (10 mL) was added with the dppp (618 mg, 1.5 mmol) solution in CH_2_Cl_2_ (10 mL) and GaCl_3_ (88 mg, 0.75 mmol). After the mixture was stirred for 6 h at room temperature, the colorless solution with a little white precipitate was obtained. After filtration, colorless block crystals were formed by the slow evaporation of the filtrate at room temperature in two days. Analysis, calculated C_54_H_52_Cl_4_P_4_GaCu: C 58.96, H 4.76%; found C 58.43, H 4.69%.

### Refinement

H atoms were positioned and refined as riding atoms with C—H = 0.93–0.97 Å and *U*_iso_(H) = 1.2*U*_eq_(C).

### Crystal data

**Table:**

[Cu(C_27_H_26_P_2_)_2_][GaCl_4_]	*F*(000) = 2256
*M**_r_* = 1099.90	*D*_x_ = 1.388 Mg m^−^^3^
Monoclinic, *P*2_1_/*n*	Mo *K*α radiation, λ = 0.71073 Å
Hall symbol: -P 2yn	Cell parameters from 2316 reflections
*a* = 21.077 (4) Å	θ = 2.3–26.6°
*b* = 11.200 (2) Å	µ = 1.28 mm^−^^1^
*c* = 22.605 (5) Å	*T* = 296 K
β = 99.424 (3)°	Block, colorless
*V* = 5264.3 (18) Å^3^	0.40 × 0.25 × 0.09 mm
*Z* = 4	

### Data collection

**Table:**

Bruker SMART APEXII CCD area-detector diffractometer	12058 independent reflections
Radiation source: fine-focus sealed tube	6644 reflections with *I* > 2σ(*I*)
Graphite monochromator	*R*_int_ = 0.052
φ and ω scans	θ_max_ = 27.5°, θ_min_ = 2.3°
Absorption correction: multi-scan (*SADABS*; Bruker, 2001)	*h* = −24→27
*T*_min_ = 0.629, *T*_max_ = 0.894	*k* = −14→14
32381 measured reflections	*l* = −29→15

### Refinement

**Table:**

Refinement on *F*^2^	Primary atom site location: structure-invariant direct methods
Least-squares matrix: full	Secondary atom site location: difference Fourier map
*R*[*F*^2^ > 2σ(*F*^2^)] = 0.055	Hydrogen site location: inferred from neighbouring sites
*wR*(*F*^2^) = 0.159	H-atom parameters constrained
*S* = 1.04	*w* = 1/[σ^2^(*F*_o_^2^) + (0.0702*P*)^2^] where *P* = (*F*_o_^2^ + 2*F*_c_^2^)/3
12058 reflections	(Δ/σ)_max_ = 0.001
577 parameters	Δρ_max_ = 0.85 e Å^−^^3^
0 restraints	Δρ_min_ = −0.76 e Å^−^^3^

### Special details

**Table:**

Geometry. All esds (except the esd in the dihedral angle between two l.s. planes) are estimated using the full covariance matrix. The cell esds are taken into account individually in the estimation of esds in distances, angles and torsion angles; correlations between esds in cell parameters are only used when they are defined by crystal symmetry. An approximate (isotropic) treatment of cell esds is used for estimating esds involving l.s. planes.
Refinement. Refinement of F^2^ against ALL reflections. The weighted R-factor wR and goodness of fit S are based on F^2^, conventional R-factors R are based on F, with F set to zero for negative F^2^. The threshold expression of F^2^ > 2sigma(F^2^) is used only for calculating R-factors(gt) etc. and is not relevant to the choice of reflections for refinement. R-factors based on F^2^ are statistically about twice as large as those based on F, and R- factors based on ALL data will be even larger.

### Fractional atomic coordinates and isotropic or equivalent isotropic displacement parameters (Å^2^)

**Table:**

	*x*	*y*	*z*	*U*_iso_*/*U*_eq_	
Cu1	0.26361 (2)	0.61161 (4)	0.99604 (2)	0.03896 (14)	
P1	0.20270 (4)	0.69588 (8)	0.91188 (5)	0.0413 (2)	
P2	0.19750 (5)	0.65483 (9)	1.06579 (5)	0.0460 (3)	
P3	0.36310 (5)	0.69750 (8)	1.02915 (5)	0.0450 (3)	
P4	0.29595 (5)	0.41400 (8)	0.99656 (5)	0.0428 (3)	
Ga2	0.49789 (3)	0.46820 (5)	0.29884 (3)	0.0752 (2)	
Cl1	0.47870 (13)	0.4662 (3)	0.38878 (12)	0.2017 (13)	
Cl2	0.59810 (7)	0.42905 (15)	0.30020 (7)	0.0989 (5)	
Cl3	0.48115 (8)	0.64747 (16)	0.26337 (13)	0.1572 (10)	
Cl4	0.43692 (9)	0.34578 (17)	0.24248 (12)	0.1529 (9)	
C1	0.15046 (19)	0.8150 (3)	0.93223 (19)	0.0516 (10)	
H1A	0.1772	0.8833	0.9458	0.062*	
H1B	0.1216	0.8391	0.8963	0.062*	
C2	0.11017 (18)	0.7853 (4)	0.98002 (19)	0.0547 (11)	
H2A	0.0916	0.7066	0.9719	0.066*	
H2B	0.0750	0.8420	0.9772	0.066*	
C3	0.1471 (2)	0.7874 (4)	1.0436 (2)	0.0599 (11)	
H3A	0.1165	0.7958	1.0710	0.072*	
H3B	0.1745	0.8575	1.0481	0.072*	
C4	0.41775 (18)	0.5977 (3)	1.0782 (2)	0.0574 (12)	
H4A	0.4604	0.6328	1.0849	0.069*	
H4B	0.4033	0.5924	1.1167	0.069*	
C5	0.42229 (18)	0.4713 (3)	1.0534 (2)	0.0580 (12)	
H5A	0.4607	0.4334	1.0748	0.070*	
H5B	0.4272	0.4769	1.0116	0.070*	
C6	0.36415 (18)	0.3912 (3)	1.05820 (18)	0.0505 (10)	
H6A	0.3498	0.4069	1.0961	0.061*	
H6B	0.3775	0.3083	1.0583	0.061*	
C11	0.24101 (18)	0.7707 (4)	0.85571 (18)	0.0496 (10)	
C12	0.2625 (2)	0.8883 (4)	0.8629 (2)	0.0743 (14)	
H12	0.2562	0.9303	0.8970	0.089*	
C13	0.2929 (3)	0.9441 (6)	0.8207 (3)	0.096 (2)	
H13	0.3054	1.0236	0.8257	0.115*	
C14	0.3042 (3)	0.8823 (7)	0.7721 (4)	0.104 (2)	
H14	0.3258	0.9193	0.7443	0.125*	
C15	0.2846 (2)	0.7664 (6)	0.7630 (2)	0.0847 (16)	
H15	0.2923	0.7252	0.7292	0.102*	
C16	0.2532 (2)	0.7110 (4)	0.8047 (2)	0.0611 (12)	
H16	0.2399	0.6322	0.7985	0.073*	
C21	0.14454 (17)	0.5972 (3)	0.86631 (17)	0.0428 (9)	
C22	0.14099 (19)	0.4790 (3)	0.88233 (19)	0.0518 (10)	
H22	0.1687	0.4493	0.9153	0.062*	
C23	0.0954 (2)	0.4032 (4)	0.8488 (2)	0.0655 (13)	
H23	0.0926	0.3237	0.8599	0.079*	
C24	0.0555 (2)	0.4459 (4)	0.8002 (2)	0.0692 (13)	
H24	0.0253	0.3958	0.7781	0.083*	
C25	0.0598 (2)	0.5641 (4)	0.7834 (2)	0.0666 (13)	
H25	0.0328	0.5930	0.7497	0.080*	
C26	0.10338 (18)	0.6381 (4)	0.81616 (18)	0.0559 (11)	
H26	0.1056	0.7175	0.8047	0.067*	
C31	0.2376 (2)	0.6914 (4)	1.14128 (19)	0.0567 (11)	
C32	0.2286 (3)	0.7988 (5)	1.1707 (2)	0.0942 (18)	
H32	0.2001	0.8555	1.1517	0.113*	
C33	0.2614 (4)	0.8213 (7)	1.2271 (3)	0.122 (3)	
H33	0.2555	0.8934	1.2459	0.147*	
C34	0.3018 (4)	0.7398 (7)	1.2553 (3)	0.115 (2)	
H34	0.3237	0.7564	1.2935	0.138*	
C35	0.3119 (3)	0.6313 (6)	1.2289 (2)	0.0916 (18)	
H35	0.3394	0.5745	1.2493	0.110*	
C36	0.2799 (2)	0.6096 (4)	1.1717 (2)	0.0649 (12)	
H36	0.2870	0.5380	1.1531	0.078*	
C41	0.13728 (18)	0.5439 (4)	1.07994 (19)	0.0514 (10)	
C42	0.1243 (2)	0.5200 (5)	1.1367 (2)	0.0769 (15)	
H42	0.1460	0.5606	1.1698	0.092*	
C43	0.0779 (3)	0.4342 (6)	1.1436 (3)	0.099 (2)	
H43	0.0699	0.4167	1.1819	0.119*	
C44	0.0448 (2)	0.3763 (5)	1.0965 (3)	0.0865 (17)	
H44	0.0141	0.3196	1.1023	0.104*	
C45	0.0561 (2)	0.4005 (4)	1.0402 (3)	0.0734 (14)	
H45	0.0326	0.3619	1.0073	0.088*	
C46	0.1030 (2)	0.4834 (4)	1.0320 (2)	0.0598 (11)	
H46	0.1113	0.4983	0.9936	0.072*	
C51	0.36291 (18)	0.8328 (3)	1.07411 (19)	0.0499 (10)	
C52	0.3953 (2)	0.8472 (4)	1.1319 (2)	0.0687 (13)	
H52	0.4202	0.7853	1.1507	0.082*	
C53	0.3906 (2)	0.9544 (4)	1.1619 (2)	0.0781 (15)	
H53	0.4123	0.9628	1.2009	0.094*	
C54	0.3552 (2)	1.0470 (4)	1.1357 (3)	0.0768 (15)	
H54	0.3523	1.1178	1.1566	0.092*	
C55	0.3237 (2)	1.0344 (4)	1.0779 (3)	0.0794 (15)	
H55	0.2998	1.0977	1.0592	0.095*	
C56	0.3272 (2)	0.9281 (4)	1.0473 (2)	0.0633 (12)	
H56	0.3053	0.9205	1.0082	0.076*	
C61	0.41146 (17)	0.7433 (3)	0.97288 (19)	0.0498 (10)	
C62	0.4624 (2)	0.8252 (4)	0.9865 (2)	0.0701 (13)	
H62	0.4724	0.8573	1.0249	0.084*	
C63	0.4974 (2)	0.8575 (4)	0.9422 (3)	0.0820 (16)	
H63	0.5305	0.9129	0.9508	0.098*	
C64	0.4840 (2)	0.8093 (5)	0.8859 (3)	0.0754 (14)	
H64	0.5080	0.8313	0.8566	0.090*	
C65	0.4352 (2)	0.7288 (5)	0.8733 (2)	0.0738 (14)	
H65	0.4261	0.6955	0.8352	0.089*	
C66	0.39951 (19)	0.6964 (4)	0.9160 (2)	0.0601 (12)	
H66	0.3664	0.6414	0.9063	0.072*	
C71	0.32982 (18)	0.3581 (4)	0.93295 (19)	0.0513 (10)	
C72	0.3667 (2)	0.2540 (5)	0.9363 (2)	0.0847 (16)	
H72	0.3719	0.2079	0.9710	0.102*	
C73	0.3957 (3)	0.2188 (7)	0.8882 (3)	0.116 (3)	
H73	0.4208	0.1502	0.8911	0.140*	
C74	0.3872 (3)	0.2847 (7)	0.8368 (3)	0.106 (2)	
H74	0.4071	0.2616	0.8048	0.127*	
C75	0.3500 (3)	0.3833 (6)	0.8322 (2)	0.0883 (17)	
H75	0.3443	0.4274	0.7968	0.106*	
C76	0.3203 (2)	0.4198 (4)	0.8791 (2)	0.0621 (12)	
H76	0.2937	0.4865	0.8745	0.075*	
C81	0.23877 (18)	0.2965 (3)	1.00825 (19)	0.0466 (9)	
C82	0.2207 (2)	0.2798 (4)	1.0643 (2)	0.0596 (11)	
H82	0.2395	0.3251	1.0969	0.072*	
C83	0.1746 (3)	0.1953 (4)	1.0710 (3)	0.0769 (14)	
H83	0.1635	0.1826	1.1086	0.092*	
C84	0.1448 (2)	0.1297 (4)	1.0230 (3)	0.0789 (16)	
H84	0.1134	0.0741	1.0280	0.095*	
C85	0.1614 (2)	0.1466 (4)	0.9687 (3)	0.0690 (14)	
H85	0.1409	0.1031	0.9360	0.083*	
C86	0.20858 (19)	0.2280 (3)	0.9610 (2)	0.0570 (11)	
H86	0.2203	0.2369	0.9233	0.068*	

### Atomic displacement parameters (Å^2^)

**Table:**

	*U*^11^	*U*^22^	*U*^33^	*U*^12^	*U*^13^	*U*^23^
Cu1	0.0391 (2)	0.0343 (2)	0.0418 (3)	0.00349 (18)	0.0020 (2)	0.0011 (2)
P1	0.0429 (5)	0.0365 (5)	0.0425 (6)	0.0022 (4)	0.0011 (4)	0.0055 (4)
P2	0.0477 (6)	0.0460 (6)	0.0441 (6)	0.0099 (4)	0.0070 (5)	−0.0008 (5)
P3	0.0404 (5)	0.0371 (5)	0.0540 (7)	0.0011 (4)	−0.0030 (5)	0.0014 (5)
P4	0.0486 (6)	0.0330 (5)	0.0459 (6)	0.0049 (4)	0.0053 (5)	0.0012 (4)
Ga2	0.0661 (3)	0.0693 (4)	0.0929 (5)	−0.0040 (3)	0.0211 (3)	−0.0132 (3)
Cl1	0.181 (2)	0.303 (3)	0.148 (2)	−0.065 (2)	0.1092 (19)	−0.041 (2)
Cl2	0.0807 (9)	0.1157 (12)	0.1036 (12)	0.0257 (8)	0.0252 (9)	0.0116 (9)
Cl3	0.0886 (11)	0.0875 (11)	0.281 (3)	0.0012 (9)	−0.0143 (15)	0.0303 (15)
Cl4	0.1225 (14)	0.1156 (14)	0.210 (2)	−0.0219 (11)	−0.0029 (15)	−0.0690 (15)
C1	0.057 (2)	0.041 (2)	0.055 (3)	0.0115 (18)	0.002 (2)	0.0033 (19)
C2	0.050 (2)	0.051 (2)	0.061 (3)	0.0162 (19)	0.003 (2)	0.000 (2)
C3	0.062 (3)	0.057 (3)	0.063 (3)	0.020 (2)	0.018 (2)	−0.001 (2)
C4	0.047 (2)	0.044 (2)	0.074 (3)	0.0046 (18)	−0.012 (2)	0.005 (2)
C5	0.047 (2)	0.050 (2)	0.073 (3)	0.0144 (19)	−0.003 (2)	0.000 (2)
C6	0.060 (2)	0.038 (2)	0.050 (2)	0.0100 (18)	0.000 (2)	0.0004 (18)
C11	0.043 (2)	0.055 (2)	0.047 (2)	−0.0014 (18)	−0.0035 (19)	0.013 (2)
C12	0.073 (3)	0.066 (3)	0.081 (4)	−0.018 (3)	0.005 (3)	0.016 (3)
C13	0.087 (4)	0.089 (4)	0.110 (5)	−0.028 (3)	0.013 (4)	0.039 (4)
C14	0.067 (3)	0.133 (6)	0.116 (6)	−0.009 (4)	0.026 (4)	0.064 (5)
C15	0.063 (3)	0.130 (5)	0.064 (3)	0.019 (3)	0.021 (3)	0.025 (4)
C16	0.053 (2)	0.073 (3)	0.057 (3)	0.001 (2)	0.010 (2)	0.017 (2)
C21	0.043 (2)	0.047 (2)	0.038 (2)	−0.0015 (16)	0.0036 (17)	0.0028 (17)
C22	0.055 (2)	0.047 (2)	0.052 (3)	0.0027 (19)	0.006 (2)	−0.0012 (19)
C23	0.073 (3)	0.046 (2)	0.075 (3)	−0.010 (2)	0.004 (3)	−0.009 (2)
C24	0.056 (3)	0.079 (3)	0.069 (3)	−0.009 (2)	0.000 (3)	−0.021 (3)
C25	0.053 (3)	0.082 (3)	0.059 (3)	−0.005 (2)	−0.009 (2)	0.003 (3)
C26	0.050 (2)	0.063 (3)	0.051 (3)	−0.0043 (19)	−0.005 (2)	0.014 (2)
C31	0.060 (3)	0.062 (3)	0.047 (3)	0.007 (2)	0.009 (2)	−0.010 (2)
C32	0.125 (5)	0.090 (4)	0.061 (3)	0.032 (3)	−0.004 (3)	−0.024 (3)
C33	0.162 (7)	0.136 (6)	0.062 (4)	0.036 (5)	−0.002 (4)	−0.034 (4)
C34	0.145 (6)	0.137 (6)	0.054 (4)	−0.020 (5)	−0.012 (4)	−0.024 (4)
C35	0.081 (4)	0.122 (5)	0.062 (3)	−0.007 (3)	−0.015 (3)	0.019 (3)
C36	0.062 (3)	0.077 (3)	0.053 (3)	0.003 (2)	0.002 (2)	0.001 (2)
C41	0.044 (2)	0.061 (3)	0.050 (3)	0.0123 (19)	0.012 (2)	0.003 (2)
C42	0.060 (3)	0.119 (4)	0.054 (3)	−0.014 (3)	0.015 (3)	0.003 (3)
C43	0.068 (3)	0.164 (6)	0.071 (4)	−0.011 (4)	0.024 (3)	0.027 (4)
C44	0.052 (3)	0.108 (4)	0.098 (5)	−0.012 (3)	0.007 (3)	0.024 (4)
C45	0.057 (3)	0.075 (3)	0.086 (4)	−0.006 (2)	0.007 (3)	0.004 (3)
C46	0.066 (3)	0.059 (3)	0.055 (3)	0.003 (2)	0.014 (2)	−0.001 (2)
C51	0.044 (2)	0.040 (2)	0.062 (3)	−0.0019 (17)	−0.001 (2)	−0.0009 (19)
C52	0.076 (3)	0.056 (3)	0.067 (3)	0.007 (2)	−0.011 (3)	−0.003 (2)
C53	0.095 (4)	0.063 (3)	0.068 (3)	−0.003 (3)	−0.010 (3)	−0.015 (3)
C54	0.086 (3)	0.054 (3)	0.090 (4)	0.000 (3)	0.013 (3)	−0.017 (3)
C55	0.087 (3)	0.043 (3)	0.105 (5)	0.013 (2)	0.007 (3)	−0.002 (3)
C56	0.069 (3)	0.048 (2)	0.069 (3)	0.010 (2)	−0.001 (3)	0.005 (2)
C61	0.038 (2)	0.045 (2)	0.063 (3)	0.0073 (17)	−0.001 (2)	0.000 (2)
C62	0.063 (3)	0.067 (3)	0.082 (4)	−0.022 (2)	0.018 (3)	−0.017 (3)
C63	0.059 (3)	0.070 (3)	0.121 (5)	−0.020 (2)	0.026 (3)	−0.008 (3)
C64	0.064 (3)	0.082 (4)	0.086 (4)	0.007 (3)	0.027 (3)	0.019 (3)
C65	0.056 (3)	0.098 (4)	0.066 (3)	0.005 (3)	0.005 (3)	−0.002 (3)
C66	0.043 (2)	0.074 (3)	0.061 (3)	−0.009 (2)	0.003 (2)	−0.003 (2)
C71	0.046 (2)	0.053 (2)	0.055 (3)	0.0011 (18)	0.010 (2)	−0.007 (2)
C72	0.094 (4)	0.086 (4)	0.074 (4)	0.039 (3)	0.013 (3)	−0.018 (3)
C73	0.098 (4)	0.148 (6)	0.099 (5)	0.053 (4)	0.000 (4)	−0.057 (5)
C74	0.080 (4)	0.164 (7)	0.078 (5)	−0.008 (4)	0.027 (4)	−0.058 (5)
C75	0.097 (4)	0.115 (5)	0.057 (3)	−0.015 (4)	0.026 (3)	−0.014 (3)
C76	0.067 (3)	0.064 (3)	0.056 (3)	−0.005 (2)	0.013 (2)	−0.002 (2)
C81	0.050 (2)	0.0335 (19)	0.055 (3)	0.0083 (17)	0.005 (2)	0.0035 (19)
C82	0.070 (3)	0.042 (2)	0.069 (3)	0.003 (2)	0.020 (3)	0.001 (2)
C83	0.091 (4)	0.064 (3)	0.083 (4)	0.003 (3)	0.039 (3)	0.006 (3)
C84	0.075 (3)	0.049 (3)	0.113 (5)	−0.010 (2)	0.018 (3)	0.015 (3)
C85	0.074 (3)	0.044 (2)	0.085 (4)	−0.007 (2)	−0.001 (3)	0.000 (2)
C86	0.067 (3)	0.036 (2)	0.065 (3)	0.0003 (19)	0.003 (2)	0.008 (2)

### Geometric parameters (Å, º)

**Table:**

Cu1—P1	2.3148 (11)	C33—C34	1.337 (8)
Cu1—P4	2.3154 (11)	C33—H33	0.9300
Cu1—P3	2.3170 (11)	C34—C35	1.386 (8)
Cu1—P2	2.3198 (12)	C34—H34	0.9300
P1—C11	1.817 (4)	C35—C36	1.378 (6)
P1—C1	1.835 (4)	C35—H35	0.9300
P1—C21	1.836 (4)	C36—H36	0.9300
P2—C31	1.822 (4)	C41—C46	1.378 (6)
P2—C41	1.842 (4)	C41—C42	1.381 (6)
P2—C3	1.847 (4)	C42—C43	1.397 (7)
P3—C51	1.825 (4)	C42—H42	0.9300
P3—C61	1.829 (4)	C43—C44	1.342 (8)
P3—C4	1.840 (4)	C43—H43	0.9300
P4—C71	1.818 (4)	C44—C45	1.360 (7)
P4—C81	1.833 (4)	C44—H44	0.9300
P4—C6	1.849 (4)	C45—C46	1.390 (6)
Ga2—Cl1	2.137 (2)	C45—H45	0.9300
Ga2—Cl4	2.1491 (18)	C46—H46	0.9300
Ga2—Cl2	2.1524 (15)	C51—C52	1.380 (6)
Ga2—Cl3	2.1694 (19)	C51—C56	1.388 (5)
C1—C2	1.516 (6)	C52—C53	1.390 (6)
C1—H1A	0.9700	C52—H52	0.9300
C1—H1B	0.9700	C53—C54	1.356 (6)
C2—C3	1.517 (6)	C53—H53	0.9300
C2—H2A	0.9700	C54—C55	1.372 (7)
C2—H2B	0.9700	C54—H54	0.9300
C3—H3A	0.9700	C55—C56	1.386 (6)
C3—H3B	0.9700	C55—H55	0.9300
C4—C5	1.531 (5)	C56—H56	0.9300
C4—H4A	0.9700	C61—C66	1.373 (6)
C4—H4B	0.9700	C61—C62	1.407 (5)
C5—C6	1.537 (5)	C62—C63	1.387 (7)
C5—H5A	0.9700	C62—H62	0.9300
C5—H5B	0.9700	C63—C64	1.368 (7)
C6—H6A	0.9700	C63—H63	0.9300
C6—H6B	0.9700	C64—C65	1.362 (7)
C11—C16	1.392 (6)	C64—H64	0.9300
C11—C12	1.393 (6)	C65—C66	1.368 (6)
C12—C13	1.383 (7)	C65—H65	0.9300
C12—H12	0.9300	C66—H66	0.9300
C13—C14	1.354 (9)	C71—C76	1.386 (6)
C13—H13	0.9300	C71—C72	1.397 (6)
C14—C15	1.367 (8)	C72—C73	1.387 (8)
C14—H14	0.9300	C72—H72	0.9300
C15—C16	1.384 (6)	C73—C74	1.365 (9)
C15—H15	0.9300	C73—H73	0.9300
C16—H16	0.9300	C74—C75	1.349 (8)
C21—C22	1.378 (5)	C74—H74	0.9300
C21—C26	1.388 (5)	C75—C76	1.378 (7)
C22—C23	1.407 (5)	C75—H75	0.9300
C22—H22	0.9300	C76—H76	0.9300
C23—C24	1.357 (6)	C81—C86	1.383 (5)
C23—H23	0.9300	C81—C82	1.394 (6)
C24—C25	1.383 (6)	C82—C83	1.382 (6)
C24—H24	0.9300	C82—H82	0.9300
C25—C26	1.363 (6)	C83—C84	1.374 (7)
C25—H25	0.9300	C83—H83	0.9300
C26—H26	0.9300	C84—C85	1.344 (7)
C31—C36	1.381 (6)	C84—H84	0.9300
C31—C32	1.402 (6)	C85—C86	1.381 (6)
C32—C33	1.370 (7)	C85—H85	0.9300
C32—H32	0.9300	C86—H86	0.9300
			
P1—Cu1—P4	121.14 (4)	C36—C31—C32	117.3 (4)
P1—Cu1—P3	116.55 (4)	C36—C31—P2	118.7 (3)
P4—Cu1—P3	98.34 (4)	C32—C31—P2	124.1 (4)
P1—Cu1—P2	99.18 (4)	C33—C32—C31	120.9 (5)
P4—Cu1—P2	113.90 (4)	C33—C32—H32	119.6
P3—Cu1—P2	107.84 (4)	C31—C32—H32	119.6
C11—P1—C1	101.16 (19)	C34—C33—C32	120.2 (6)
C11—P1—C21	102.58 (18)	C34—C33—H33	119.9
C1—P1—C21	101.84 (18)	C32—C33—H33	119.9
C11—P1—Cu1	120.82 (12)	C33—C34—C35	121.6 (6)
C1—P1—Cu1	111.41 (14)	C33—C34—H34	119.2
C21—P1—Cu1	116.42 (12)	C35—C34—H34	119.2
C31—P2—C41	102.5 (2)	C36—C35—C34	118.2 (5)
C31—P2—C3	103.4 (2)	C36—C35—H35	120.9
C41—P2—C3	101.98 (19)	C34—C35—H35	120.9
C31—P2—Cu1	116.47 (14)	C35—C36—C31	121.8 (5)
C41—P2—Cu1	119.01 (14)	C35—C36—H36	119.1
C3—P2—Cu1	111.45 (15)	C31—C36—H36	119.1
C51—P3—C61	101.95 (19)	C46—C41—C42	118.5 (4)
C51—P3—C4	103.15 (18)	C46—C41—P2	118.9 (3)
C61—P3—C4	102.94 (19)	C42—C41—P2	122.6 (4)
C51—P3—Cu1	116.12 (13)	C41—C42—C43	119.1 (5)
C61—P3—Cu1	118.01 (13)	C41—C42—H42	120.5
C4—P3—Cu1	112.72 (13)	C43—C42—H42	120.5
C71—P4—C81	102.67 (19)	C44—C43—C42	121.8 (5)
C71—P4—C6	101.01 (19)	C44—C43—H43	119.1
C81—P4—C6	103.92 (18)	C42—C43—H43	119.1
C71—P4—Cu1	118.60 (14)	C43—C44—C45	119.9 (5)
C81—P4—Cu1	119.15 (12)	C43—C44—H44	120.1
C6—P4—Cu1	109.15 (12)	C45—C44—H44	120.1
Cl1—Ga2—Cl4	111.68 (11)	C44—C45—C46	119.7 (5)
Cl1—Ga2—Cl2	108.81 (10)	C44—C45—H45	120.2
Cl4—Ga2—Cl2	111.63 (8)	C46—C45—H45	120.2
Cl1—Ga2—Cl3	108.46 (12)	C41—C46—C45	121.1 (5)
Cl4—Ga2—Cl3	109.03 (9)	C41—C46—H46	119.4
Cl2—Ga2—Cl3	107.08 (7)	C45—C46—H46	119.4
C2—C1—P1	116.6 (3)	C52—C51—C56	118.2 (4)
C2—C1—H1A	108.1	C52—C51—P3	125.3 (3)
P1—C1—H1A	108.1	C56—C51—P3	116.5 (3)
C2—C1—H1B	108.1	C51—C52—C53	119.9 (4)
P1—C1—H1B	108.1	C51—C52—H52	120.0
H1A—C1—H1B	107.3	C53—C52—H52	120.0
C1—C2—C3	114.3 (3)	C54—C53—C52	121.8 (5)
C1—C2—H2A	108.7	C54—C53—H53	119.1
C3—C2—H2A	108.7	C52—C53—H53	119.1
C1—C2—H2B	108.7	C53—C54—C55	118.8 (5)
C3—C2—H2B	108.7	C53—C54—H54	120.6
H2A—C2—H2B	107.6	C55—C54—H54	120.6
C2—C3—P2	115.3 (3)	C54—C55—C56	120.5 (4)
C2—C3—H3A	108.4	C54—C55—H55	119.8
P2—C3—H3A	108.4	C56—C55—H55	119.8
C2—C3—H3B	108.4	C55—C56—C51	120.8 (4)
P2—C3—H3B	108.4	C55—C56—H56	119.6
H3A—C3—H3B	107.5	C51—C56—H56	119.6
C5—C4—P3	114.3 (3)	C66—C61—C62	118.1 (4)
C5—C4—H4A	108.7	C66—C61—P3	120.4 (3)
P3—C4—H4A	108.7	C62—C61—P3	121.5 (4)
C5—C4—H4B	108.7	C63—C62—C61	119.3 (5)
P3—C4—H4B	108.7	C63—C62—H62	120.4
H4A—C4—H4B	107.6	C61—C62—H62	120.4
C4—C5—C6	114.6 (3)	C64—C63—C62	121.0 (5)
C4—C5—H5A	108.6	C64—C63—H63	119.5
C6—C5—H5A	108.6	C62—C63—H63	119.5
C4—C5—H5B	108.6	C65—C64—C63	119.3 (5)
C6—C5—H5B	108.6	C65—C64—H64	120.3
H5A—C5—H5B	107.6	C63—C64—H64	120.3
C5—C6—P4	113.4 (3)	C64—C65—C66	120.8 (5)
C5—C6—H6A	108.9	C64—C65—H65	119.6
P4—C6—H6A	108.9	C66—C65—H65	119.6
C5—C6—H6B	108.9	C65—C66—C61	121.5 (4)
P4—C6—H6B	108.9	C65—C66—H66	119.3
H6A—C6—H6B	107.7	C61—C66—H66	119.3
C16—C11—C12	116.8 (4)	C76—C71—C72	117.6 (4)
C16—C11—P1	121.4 (3)	C76—C71—P4	120.3 (3)
C12—C11—P1	121.8 (4)	C72—C71—P4	122.1 (4)
C13—C12—C11	121.7 (6)	C73—C72—C71	120.4 (6)
C13—C12—H12	119.2	C73—C72—H72	119.8
C11—C12—H12	119.2	C71—C72—H72	119.8
C14—C13—C12	119.5 (6)	C74—C73—C72	120.1 (6)
C14—C13—H13	120.2	C74—C73—H73	119.9
C12—C13—H13	120.2	C72—C73—H73	119.9
C13—C14—C15	121.2 (6)	C75—C74—C73	120.1 (6)
C13—C14—H14	119.4	C75—C74—H74	120.0
C15—C14—H14	119.4	C73—C74—H74	120.0
C14—C15—C16	119.4 (6)	C74—C75—C76	121.0 (6)
C14—C15—H15	120.3	C74—C75—H75	119.5
C16—C15—H15	120.3	C76—C75—H75	119.5
C15—C16—C11	121.5 (5)	C75—C76—C71	120.6 (5)
C15—C16—H16	119.3	C75—C76—H76	119.7
C11—C16—H16	119.3	C71—C76—H76	119.7
C22—C21—C26	118.6 (4)	C86—C81—C82	117.9 (4)
C22—C21—P1	119.4 (3)	C86—C81—P4	121.2 (3)
C26—C21—P1	122.0 (3)	C82—C81—P4	120.8 (3)
C21—C22—C23	120.0 (4)	C83—C82—C81	119.6 (5)
C21—C22—H22	120.0	C83—C82—H82	120.2
C23—C22—H22	120.0	C81—C82—H82	120.2
C24—C23—C22	120.1 (4)	C84—C83—C82	121.2 (5)
C24—C23—H23	119.9	C84—C83—H83	119.4
C22—C23—H23	119.9	C82—C83—H83	119.4
C23—C24—C25	120.0 (4)	C85—C84—C83	119.6 (5)
C23—C24—H24	120.0	C85—C84—H84	120.2
C25—C24—H24	120.0	C83—C84—H84	120.2
C26—C25—C24	120.0 (4)	C84—C85—C86	120.5 (5)
C26—C25—H25	120.0	C84—C85—H85	119.7
C24—C25—H25	120.0	C86—C85—H85	119.7
C25—C26—C21	121.3 (4)	C85—C86—C81	121.3 (5)
C25—C26—H26	119.4	C85—C86—H86	119.4
C21—C26—H26	119.4	C81—C86—H86	119.4

### Hydrogen-bond geometry (Å, º)

Cg1 and Cg2 are the centroids of the C21–C26 and C81–C86 benzene rings, respectively.

**Table:**

*D*—H···*A*	*D*—H	H···*A*	*D*···*A*	*D*—H···*A*
C14—H14···*Cg*1^i^	0.93	2.77	3.702 (8)	175
C55—H55···*Cg*2^ii^	0.93	2.66	3.526 (5)	155

Symmetry codes: (i) −*x*+1/2, *y*+1/2, −*z*+3/2; (ii) *x*, *y*+1, *z*.
